# Pharmacokinetics and tissue residues of albendazole sulphoxide and its metabolites in donkey after intramuscular injection

**DOI:** 10.1002/vms3.1393

**Published:** 2024-04-19

**Authors:** Zaijian Li, Xuling Qin, Changfa Wang, Wenqiang Liu

**Affiliations:** ^1^ College of Agricultural Science and Engineering Liaocheng University Liaocheng Shandong P. R. China

**Keywords:** albendazole sulphoxide, donkey, metabolites, pharmacokinetics, residues

## Abstract

**Background:**

Various anti‐parasitic drugs are used to control donkey parasitic diseases. The abuse of donkey drugs leads to the disposition of residues in the edible parts of treated donkeys.

**Objectives:**

The aim of this study was to (1) analyse the pharmacokinetics of ABZSO to serve as reference for the dosage regimen in donkey; and (2) calculate the withdrawal times of the ABZSO in the tissue of the donkey.

**Methods:**

The concentrations of ABZSO and its metabolites in plasma and tissues were determined using high‐performance liquid chromatography with an ultraviolet detector. Pharmacokinetic analysis was performed by the programme 3p97.

**Results:**

The plasma concentrations of ABZSO and ABZSO_2_ concentration–time data in donkey conformed to the absorption one‐compartment open model. The t1/2ke of ABZSO was 0.67 h, whereas the *t*_1/2_ *_k_*_e_ was 12.93 h; the *C*_max_ and the *T*_p_ were calculated as 0.58 μg mL^−1^ and 3.01 h. The *V*_d/F_ of ABZSO was estimated to be 10.92 L kg^−1^; the area under the curve (AUC) was 12.81 μg mL^−1^ h. The *C*_max_ and AUC values of ABZSO were higher than those of ABZSO_2_; however, *t*_1/2_ *_K_*_e_ and *V*_d/F_ were lower. Other pharmacokinetics parameters were similar between the two metabolites.

**Conclusions:**

The results revealed that ABZSO_2_ was the main metabolite of ABZSO in donkey plasma. The concentrations of ABZSO and its chief metabolite (ABZSO_2_) were detected in liver, kidney, skin and muscle; however, ABZ‐SO_2_NH_2_ was only detected in liver and kidney. The results also revealed that the depletion of ABZSO and its metabolite in donkey was longer, especially in skin.

## Graphical abstract

The results revealed that ABZSO_2_ was the main metabolite of ABZSO in donkey plasma. The concentrations of ABZSO and its cheif metabolite (ABZSO_2_) were detected in liver, kidney, skin and muscle; however, ABZ‐SO_2_NH_2_ was only detected in liver and kidney.

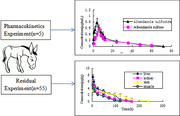

## 1 INTRODUCTION

Donkey farming is now an important industry in North China. However, donkey disease is becoming a serious problem. Various anti‐parasitic drugs are used to control donkey parasitic diseases. The abuse of donkey drugs leads to the disposition of residues in the edible parts of treated donkeys (Imani‐Baran et al., 2020). Benzimidazol anthelmintics have been used in donkeys for the treatment of various parasitic diseases because of their good anti‐parasite activity and relative minor toxicity (Bhavsar et al., 2020). Benzimidazol anthelmintics are broad‐spectrum anti‐parasitic agents used against the most important animal gastrointestinal helminth species (Son et al., 2020). The main metabolites of albenzdazole (ABZ) were albendazole sulphoxide (ABZSO), albendazole sulfone (ABZSO_2_) and albendazole‐2‐aminosulfone (ABZ‐SO_2_NH_2_). ABZSO is the main anthelmintically active metabolite recovered in blood (Theurillat et al., 2021; Zhang et al., 2020) and tissues (Busatto et al., 2018; Pereira Cordeiro et al., 2021), after treatment with ABZ in cattle and sheep. Pharmacokinetics of ABZSO has been extensively studied in other species, including humans (Ceballos et al., 2018; Myers et al., 2021; Paredes et al., 2018). Donkey is one of the most important herbivores in the world, and it is easy to suffer from parasitic disease. Chemotherapy is widely used to control Mammalian parasitic infection. ABZSO is an available anthelmintic compound in ruminant species as an oral preparation and, more recently, as an injectable formulation in cattle (Feng et al., 2017). Now it is also gradually used to control donkey parasitic diseases. Lixin have studied the mice infected with *Echinococcus granulosus* treatment for ABZSO, and the results showed that ABZ is secondary to ABZSO in the anthelmintic efficacy. The reason is that the solubility of HCL‐ABZSO is greater than ABZ in intestinal and then has good absorption (Pavan et al., 2007). ABZSO is allowed to be used for the prevention and treatment of parasitic diseases in donkeys in China. So HCL‐ABZSO would have a good prospect of application in donkey.

As the use of benzimidazol anthelmintics in animal production industries has been increasing over the last two decades, what is more, the problems which the abuse of drugs leads to residues have become serious. However, toxicological studies in both farm and laboratory animals have shown ABZ and ABZSO could be teratogenic (Belew et al., 2021; Portela et al., 2020). ‘Maximum Residue Limits for Veterinary Drugs in Animal Food in China’ stipulates the total amount of ABZSO, ABZSO_2_ and ABZ‐SO_2_NH_2_ as residual markers of ABZ in animal food (muscle, fat: 100 μg kg^−1^; liver, kidney: 5000 μg kg^−1^). In order to guide the use of HCL‐ABZSO for the treatment of donkey disease in the farming of donkey, information was needed on the pharmacokinetics in donkey. Nevertheless, no data is available on the pharmacokinetics of HCL‐ABZSO in donkey. Studies on the pharmacokinetics of HCL‐ABZSO were performed in cattle, horse and fish (Dar et al., 2017; Li et al., 2012; Myers et al., 2021). As the parameters of pharmacokinetics and residues are variable in different species, it is unreasonable that HCL‐ABZSO data is used directly in donkey.

This study was undertaken to research pharmacokinetics and tissue residues of HCL‐ABZSO in donkey. The objectives of this study are (1) to analyse the pharmacokinetics of ABZSO, to serve as reference for the dosage regimen in donkey; (2) to calculate the withdrawal times (WDTs) of the ABZSO in the tissue of the donkey.

## 2 MATERIALS AND METHODS

### 2.1 Ethics statement and facility

The animal research were carried out in accordance with the recommendations in the Guide for the Care and Use of Laboratory Animals of the Ministry of Science and Technology of the People's Republic of China. The protocols for donkey studies were approved by the Committee on the Ethics of Animal Experiments of Liaocheng University. The ethics approval numbers are 2023022732.

### 2.2 Chemicals

All agents used for the analysis were of the high‐performance liquid chromatography (HPLC) grade. HCL‐ABZSO with a chemical purity of 98.0% (No. 20201001) was purchased from Huashu. ABZSO_2_ and ABZ‐SO_2_NH_2_ with a chemical purity of 98.1% (No. 200606) were obtained from Kanglong. All other organic solvents were of analytical grade.

### 2.3 Animals

Sixty healthy donkeys (Dezhou donkeys, 30 males, 30 females, 2 years old) were used for this study. Average body‐weight (BW) was 100 kg. Five donkeys were used for pharmacokinetics experiment, and 55 animals were used for residues experiment. The donkeys were fed antibiotic‐free feed (silage corn) twice a day, and water was available. All animals remained in good health during the experiments.

### 2.4 Drug administration and sampling

During the study of pharmacokinetics by IM injection, donkeys were given a single dose of 7.5 mg kg^−1^ BW ABZSO of a solution with dimethyl sulphoxide. In the residues study, donkeys were given a multi‐dose of 7.5 mg kg^−1^ BW for 3 consecutive days by IM injection. After IM injection administration in the study of pharmacokinetics, five donkeys were sampled at each time‐point (Table 1), using evacuated syringes containing heparin to prevent clotting. The plasma was stored at −20°C.

**TABLE 1:** ABZSO and its metabolites concentrations in plasma following IM injection (*n* = 5).

	Mean drug concentration (μg mL^−1^) ± S.D.	
Time (h)	ABZSO	ABZSO_2_
0.25	0.11 ± 0.02	ND
0.5	0.23 ± 0.04	ND
1	0.47 ± 0.05	0.08 ± 0.03
2	0.55 ± 0.01	0.26 ± 0.07
4	0.78 ± 0.14	0.41 ± 0.08
6	0.59 ± 0.12	0.58 ± 0.05
8	0.47 ± 0.09	0.37 ± 0.06
10	0.41 ± 0.07	0.28 ± 0.06
12	0.28 ± 0.06	0.21 ± 0.04
24	0.16 ± 0.05	0.15 ± 0.04
36	0.11 ± 0.04	0.11 ± 0.03
48	0.06 ± 0.02	0.05 ± 0.02
60	0.03 ± 0.01	0.03 ± 0.01
72	ND	ND

Abbreviations: ND, no detection; S.D., standard deviation.

For the purpose of the residue study of ABZSO, tissues, such as liver, muscle, kidney and skin, were sampled at 4, 8, 12, 24, 48, 72, 96, 120, 144, 168 and 240 h after the last administration. The tissues of donkey were collected at each time‐point. Control samples (*n* = 5) were collected alternately from the control groups. All samples were stored at −20°C.

### 2.5 Sample analysis

The HPLC system was Shimadzu LC‐2030 series equipment, with a variable wavelength detector. ABZSO and its metabolite detection were performed at 290 nm. An automatic injection of 20 μL was performed on SB‐C_18_ stainless steel column (250 × 4.6 mm^2^, 5 μm). Thermostatted column compartment was used to maintain the column temperature at 32°C. The mobile phase consisted of acetonitrile and 0.02 mol L^−1^ ammonium acetate solution (pH = 4.8) (24:76, v/v), and the flow rate was 1.0 mL min^−1^.

After samples were thawed at room temperature, the extraction of ABZSO and its metabolites in plasma and tissues was carried out. A sample of 4 mL plasma was placed in a 15 mL plastic centrifuge tube. A 0.4 mol L^−1^ sodium hydroxide solution of 1000 μL and 10 mL of ethylacetate used as extractant were added; the mixture was vortexed for 2 min and then centrifuged for 10 min at 3000 rpm. The supernatant was removed to a fresh tube and dried at 70°C under nitrogen. Extraction of ABZSO and its metabolites in muscle, skin, liver and kidney samples was carried out according to the method of plasma analysis. The homogenate (4.0 g) was transferred to a 15 mL plastic centrifuge tube. The samples were extracted three times using ethylacetate (10 mL) in order to achieve high recovery. The extracts were combined and dried at 70°C under nitrogen. The residue was reconstituted in 200 μL of the mobile phase and then degreased by *N*‐hexane before analysis.

### 2.6 Method validation

The method was validated for plasma, muscle, skin, liver and kidney tissues. The standard calibration curves were prepared within the concentrations of 0.05, 0.1, 0.2, 0.5, 2 and 5 μg mL^−1^ in plasma and 0.05, 0.1, 0.5, 1, 5 and 10 μg g^−1^ in all tissues. Linearity, recovery, intra‐ and inter‐assay precisions and accuracies were determined by the standard curve. Recovery was calculated by comparing the peak area of the drugs from processed samples with that from the drug standard in the mobile phase. Accuracy was determined by comparing the measured concentration to its true value. The variability in the peak area ratios at each concentration was determined as an indicator of the precision. The limit of detection (LOD) and the limit of quantitation of the drugs were defined as the drug concentrations resulting in a peak height 3 and 10 times the signal noise, respectively.

### 2.7 Pharmacokinetic and residue analysis

Pharmacokinetic analysis was performed by the programme 3p97 (version 1.0, edited by the Chinese Pharmacological Society). Following a single dose of an IM injection administration, the pharmacokinetic parameters of ABZSO and ABZSO_2_ were both calculated according to the one‐compartment open models with first‐order rate processes, using the following equation:

$$C=M(e^{-k_{e}t}-e^{-K_{a}t})$$

where *C* is the plasma concentration at any time (*t*); *M* is zero time intercept of the elimination phase; *k*_e_ is the elimination rate constant; *K*_a_ is the absorption rate constant.

The area under the curve (AUC) was determined according to the following equations: AUC (μg mL^−1 ^h) = M (1/*K*_e_–1/*K*_a_)

The volume of distribution was calculated from the equation:

$$V_{d}/F=X_{0}/\left(\mathrm{AUC}K_{e}\right)$$

Total body clearance (CL_b_) was calculated using the following equations:

$$CL_{b}\left(1\;h^{-1}\;kg^{-1}\right)=V_{d}K_{e}$$

The elimination half‐life (t1/2ke) was determined by the following equation:

$$t_{1/2\mathrm{Ke}}=0.693/K_{e}$$

In the residues study, the elimination characteristic of the drug from each tissue was estimated according to first‐order rate processes using the equation: C = C_0_e^−^ *^kt^*, where C is the tissue concentrations of ABZSO and its metabolites at time t; C_0_ is an extrapolate concentration of ABZSO and its metabolites in target tissues after IM injection; *k is* the elimination rate constant. The data was analysed by the least squares method. The elimination half‐life (t1/2ke) was calculated from the equation: *t*_1/2_ *_k_*_e_ = 0.693/k. The WDT would be calculated by WT1.4 software according to GB 31650‐2019, which formulated the residue marker for ABZ in food animals (ABZ‐SO_2_NH_2_) and the maximum residue limit (muscle: 100 μg kg^−1^; fat: 100 μg kg^−1^; liver: 5000 μg kg^−1^; kidney: 5000 μg kg^−1^).

## 3 RESULTS

### 3.1 Method validation

The values of correlation coefficient (*r*), recovery, accuracy, precision of inter‐ and intra‐days and LOD of the analytical method for ABZSO and its metabolites in all tissues are listed in Table 2.

**TABLE 2:** The method validation in plasma, muscle, liver, akin and kidney.

Tissue	Recovery (%)	Precision (%)		LOD (μg g^−1^)		
		Intra‐day	Inter‐day	ABZSO	ABZSO_2_	ABZ‐SO_2_NH_2_
Plasma	88.98–91.54	<3.58	<5.78	0.01	0.01	0.02
Muscle	80.02–81.68	<4.89	<8.79	0.02	0.02	0.02
Skin	72.45–75.85	<4.55	<9.81	0.02	0.02	0.02
Liver	74.38–76.82	<4.78	<7.67	0.02	0.05	0.02
Kidney	76.82–77.93	<7.72	<8.45	0.02	0.02	0.02

*Note*: LOD, defined as concentration of drug resulting in a peak height three times the signal noise.

### 3.2 Pharmacokinetics

**FIGURE 1:**
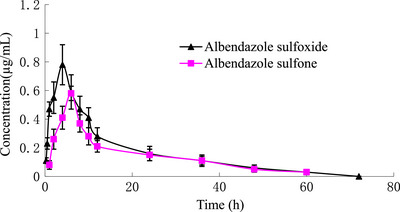
Albendazole sulphoxide (ABZSO) and albendazole sulfone (ABZSO_2_) levels in plasma of donkey after a single IM administration (7.5 mg kg^−1^).

ABZSO and ABZSO_2_ were recovered in plasma between 0.5 and 72 h after ABZSO IM injection administration. ABZ‐SO_2_NH_2_ was not detected in plasma at any time. Pharmacokinetic parameters of ABZSO and its chief metabolite (ABZSO_2_) in donkey obtained after IM injection with HCL‐ABZSO of 7.5 mg kg^−1^ are shown in Table 3. The plasma concentrations of ABZSO and ABZSO_2_ concentration–time data in donkey conformed to the absorption one‐compartment open model. The maximum plasma concentration (*C*_max_) of ABZSO with 0.58 μg mL^−1^ was obtained at 3.01 h, compared to ABZSO_2_ (0.43 μg mL^−1^ at 3.14 h), respectively. The absorption half‐lives (t1/2ke) of ABZSO were calculated to be 0.67 h, whereas the elimination half‐lives (t1/2ke) was 12.93 h. The distribution volume (V_d_/F) of ABZSO was estimated to be 10.92 L kg^−1^, whereas that of ABZSO_2_ was 15.07 L kg^−1^. The total clearance (CL_b_) of ABZSO was computed as 0.59 L (h kg)^−1^, and that of ABZSO_2_ was 0.72 L (h kg)^−1^. The area under the concentration–time curve (AUC) of ABZSO was 12.81 63.12 μg mL^−1 ^h, compared to ABZSO_2_ (10.38 μg mL^−1^), respectively. Plasma concentration of ABZSO and ABZSO_2_ vs. time curve detected in donkey is given in Figure 1.

**TABLE 3:** Pharmacokinetic parameters of ABZSO and its ABZSO_2_ in donkey following IM injection (*n* = 5).

Parameters	Units	ABZSO	ABZSO_2_
A	μg mL^−1^	0.73 ± 0.12	0.52 ± 0.09
*K*_e_	1 h	0.054 ± 0.09	0.05 ± 0.01
*K*_a_	1 h	1.04 ± 0.11	1.02 ± 0.14
t1/2ke	h	0.67 ± 0.08	0.68 ± 0.12
*t*_1/2_ *_K_*_e_	h	12.93 ± 1.42	14.46 ± 1.96
*T*_max_	h	3.01 ± 0.43	3.14 ± 0.53
*C*_max_	μg mL^−1^	0.58 ± 0.11	0.43 ± 0.05
V_d_/F	L kg^−1^	10.92 ± 1.76	15.07 ± 1.87
AUC	μg mL^−1^h	12.81 ± 1.73	10.38 ± 1.64
CL_b_	L (h kg)^−1^	0.59 ± 0.08	0.72 ± 0.13

Abbreviations: AUC, area under curve; CL_b_, total body clearance of the drug; *C*_max_, the maximum plasma concentration; Ka, absortion rate constant; Ke, elimination rate constant; *t*_1/2*k*a_, absorption half‐life of the drug; *t*_1/2_ *_K_*_e_, elimination half‐life of the drug; *T*_max_, the time‐point of maximum plasma concentration; V_d_/F, extensive apparent volume of the central compartment.

### 3.3 Residues

**FIGURE 2:**
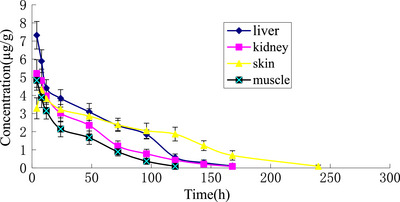
Albendazole sulphoxide (ABZSO) and its metabolites in all tissues of donkey after the last dosing.

ABZSO and its metabolite concentrations in liver, kidney, skin and muscle are listed in Tables 4, 5, 6, 7, respectively. It was obvious that the t1/2ke in skin was longer than that in other tissues. ABZSO and ABZSO_2_ were detected in all tissues. However, ABZSO_2_‐NH_2_ was only detected in liver and kidney, which was not detected in muscle and skin. The concentration of ABZSO and its metabolites was found to be higher in liver than that in other tissues. However, it decreased rapidly in liver after the 24 h post‐administration. ABZSO and its metabolite concentration–time curves in all tissues are given in Figure 2.

**TABLE 4:** ABZSO and its metabolites concentration in liver (*n* = 5).

Time (h)	Mean ABZSO and its metabolites concentration (μg g^−1^) ± S.D. in liver			
	ABZSO	ABZSO_2_	ABZ‐SO_2_NH_2_	Total concentration
4	4.93 ± 0.98	2.39 ± 0.12	ND	7.32 ± 0.86
8	4.26 ± 1.05	1.49 ± 0.33	0.14 ± 0.05	5.89 ± 0.68
12	3.56 ± 0.87	0.60 ± 0.17	0.25 ± 0.08	4.41 ± 0.41
24	2.98 ± 0.85	0.47 ± 0.12	0.39 ± 0.13	3.84 ± 0.39
48	2.22 ± 0.73	0.38 ± 0.09	0.49 ± 0.15	3.09 ± 0.43
72	1.67 ± 0.36	0.32 ± 0.12	0.35 ± 0.08	2.34 ± 0.38
96	1.32 ± 0.27	0.25 ± 0.06	0.28 ± 0.06	1.85 ± 0.35
120	0.23 ± 0.08	0.17 ± 0.05	0.18 ± 0.04	0.58 ± 0.29
144	0.10 ± 0.04	0.12 ± 0.03	0.07 ± 0.02	0.29 ± 0.18
168	ND	0.07 ± 0.02	0.04 ± 0.02	0.11 ± 0.11
240	ND	ND	ND	ND

Abbreviations: ND, no detection; S.D., standard deviation.

**TABLE 5:** ABZSO and its metabolites concentration in kidney in kidney (*n* = 5).

Time (h)	Mean ABZSO and its metabolites concentration (μg g^−1^) ± S.D. in kidney			
	ABZSO	ABZSO_2_	ABZ‐SO_2_NH_2_	Total concentration
4	4.84 ± 0.56	0.21 ± 0.11	ND	5.21 ± 0.76
8	4.15 ± 1.06	0.41 ± 0.25	0.29 ± 0.12	4.82 ± 0.63
12	3.41 ± 0.82	0.34 ± 0.74	0.22 ± 0.09	3.97 ± 0.46
24	2.65 ± 0.09	0.19 ± 0.08	0.18 ± 0.11	3.02 ± 0.48
48	2.11 ± 0.43	0.13 ± 0.05	0.11 ± 0.05	2.35 ± 0.47
72	1.08 ± 0.34	0.07 ± 0.02	0.06 ± 0.02	1.21 ± 0.28
96	0.69 ± 0.17	0.05 ± 0.02	0.04 ± 0.02	0.78 ± 0.25
120	0.37 ± 0.07	0.04 ± 0.01	ND	0.43 ± 0.19
144	0.19 ± 0.05	0.02 ± 0.01	ND	0.21 ± 0.15
168	0.08 ± 0.03	ND	ND	0.08 ± 0.03
240	ND	ND	ND	ND

Abbreviations: ND, no detection; S.D., standard deviation.

**TABLE 6:** ABZSO and its metabolites concentration in skin (*n* = 5).

Time (h)	Mean ABZSO and its metabolites concentration (μg g^−1^) ± S.D. in skin			
	ABZSO	ABZSO_2_	ABZ‐SO_2_NH_2_	Total concentration
4	2.89 ± 0.65	0.40 ± 0.12	ND	3.29 ± 0.58
8	3.49 ± 1.25	0.83 ± 032	ND	4.32 ± 0.51
12	3.17 ± 0.57	0.66 ± 0.17	ND	3.83 ± 0.49
24	2.80 ± 1.24	0.43 ± 0.15	ND	3.23 ± 0.47
48	2.51 ± 0.72	0.35 ± 0.07	ND	2.86 ± 0.41
72	2.09 ± 0.46	0.29 ± 0.08	ND	2.38 ± 0.36
96	1.83 ± 0.47	0.22 ± 0.08	ND	2.05 ± 0.42
120	1.69 ± 0.36	0.18 ± 0.05	ND	1.87 ± 0.38
144	1.10 ± 0.28	0.13 ± 0.04	ND	1.23 ± 0.27
168	0.62 ± 0.14	0.07 ± 0.02	ND	0.69 ± 0.26
240	0.09 ± 0.03	ND	ND	0.09 ± 0.07

Abbreviations: ND, no detection; S.D., standard deviation.

**TABLE 7:** ABZSO and its metabolites concentration in muscle (*n* = 5).

Time (h)	Mean ABZSO and its metabolites concentration (μg g^−1^) ± S.D. in muscle			
	ABZSO	ABZSO_2_	ABZ‐SO_2_NH_2_	Total concentration
4	3.89 ± 0.54	0.94 ± 0.24	ND	4.83 ± 0.54
8	3.17 ± 0.65	0.72 ± 0.25	ND	3.89 ± 0.56
12	2.69 ± 0.67	0.46 ± 0.62	ND	3.15 ± 0.45
24	1.84 ± 0.45	0.31 ± 0.15	ND	2.15 ± 0.43
48	1.41 ± 0.37	0.26 ± 0.07	ND	1.67 ± 0.37
72	0.70 ± 0.18	0.19 ± 0.05	ND	0.89 ± 0.23
96	0.25 ± 0.06	0.12 ± 0.04	ND	0.37 ± 0.09
120	0.07 ± 0.03	0.02 ± 0.02	ND	0.09 ± 0.05
144	ND	ND	ND	ND
168	ND	ND	ND	ND
240	ND	ND	ND	ND

Abbreviations: ND, no detection; S.D., standard deviation.

The results reveal that the WDT was 3 days calculated by WT1.4 software according to GB 31650‐2019. Compared with the other tissues, the concentrations of ABZSO and its metabolites were higher in liver at each time‐point, whereas the depletion of ABZSO and its metabolite in skin was longer. Because skin is the edible product of donkey, which is the main raw material of donkey‐hide gelatin. The elimination of ABZSO and its metabolites in skin was the slowest, which behaved as a reservoir in donkey. The WDT of ABZSO in donkey should not be less than 3 days when administered by IM injection with a multi‐dose of 7.5 mg kg^−1^ BW.

## 4 DISCUSSION

After IM injection administration, ABZSO was rapidly absorbed in donkey. The *K*_a_ of ABZSO was 1.04 h^−1^, which is more rapidly than ABZ directly. Goudah (2003) studied the pharmacokinetics of ABZ and ABZSO in sheep after oral administration. These results showed that the absorption of two drugs is similar, whereas the elimination rate of ABZSO was faster than the ABZ. The data in this experiment was consistent with Goudah's studies. This may be due to its good solubility and easy absorption; however, other benzimidazole anthelmintic solutes lowly and absorps poorly in the intestine.

The drug was in the form of ABZSO mainly, whereas the form of ABZSO_2_ is secondary in the plasma, which may be because only a small amount of ABZSO converted into ABZSO_2_ and then metabolized to ABZ‐SO_2_NH_2_. (Capece et al., 2000) have studied the pharmacokinetics of ABZ and ABZSO in sheep and goats; Formentini et al. (2001) have reported ABZ and ABZSO kinetic dispositions after treatment with different formulations in calves. The results demonstrated that the concentration of ABZSO in plasma was higher than that of ABZSO_2_ in these two kinds of animals, which was in accordance with the conclusion of donkey. There was little difference between ABZSO and ABZSO_2_ about the peak time (*T*_p_) in donkey, which was consistent with *T*p in scalper.

The V_d_/F of ABZSO was 10.92 L kg^−1^, and which of its main metabolite (ABZSO_2_) was 15.07 L kg^−1^. It was assessed that ABZSO and ABZSO_2_ were well distributed throughout the body. Because the parasite was generally located in donkey skin, the distribution characteristic in skin could be considered an advantage of ABZSO. So ABZSO, in treating donkey parasitic disease, would have good prospect.

There was no significant deviation between ABZSO and ABZSO_2_ about the t1/2ke in donkey. The t1/2ke of ABZSO was a little shorter (12.93 h) than that of ABZSO_2_ (14.46). The speed of absorption was rapid, whereas the speed of elimination was slow. The CL_b_ of ABZSO and ABZSO_2_ also had no significant deviation, which was 0.59 and 0.72 L (h kg)^−1^, respectively.

ABZSO and its metabolite residues were detected in the tissue of the donkey, but GB 31650‐2019 formulated the residue marker for ABZ in food animals as ABZ‐SO_2_NH_2_. It was indicated that the t1/2ke of ABZSO and its metabolites in skin was slower than that in other tissues (*p* < 0.01). This may be related to the accumulation of drugs in the skin (Benigni et al., 2018). The results indicated that ABZSO_2_ was the main metabolite of ABZSO; ABZSO and its main metabolite (ABZSO_2_) were detected in liver, kidney, muscle and skin, whereas ABZ‐SO_2_NH_2_ was only detected in liver and kidney, this is in accordance with other mammals (Mortezaei et al., 2019; Pérez‐Brígido et al., 2020). The drug in all tissues was eliminated slowly, which could still be detected at 240 h time‐point after IM injection. The concentration of ABZSO and its metabolites was highest in skin at this time‐point, compared to other tissues. The elimination of ABZSO and its metabolites in donkey skin was slowest, which behaved as a reservoir. Skin is the edible product of donkey, which is the raw material of donkey‐hide gelatin, although the residue marker for ABZ in food animals is ABZ‐SO_2_NH_2_, which was not detected in skin.

Based on the results of the present study, it can be concluded that the elimination of ABZSO and its metabolites in donkey skin was slower than in other tissues, which was in accordance with the research of ABZSO in other mammals. To guarantee that tissue samples are safe for consumption, it is suggested that the WDT of ABZSO in donkey should not be less than 3 days.

## AUTHOR CONTRIBUTIONS

Zaijian Li and Wenqiang Liu designed this study and revised the experiments. Changfa Wang managed the whole experimental process. Xuling Qin helped with sampling and analyte detection. All authors have read and approved the final manuscript.

## CONFLICT OF INTEREST STATEMENT

We declare that we have no financial and personal relationships with other people or organizations that can inappropriately influence our work, and there is no professional or other personal interest of any nature or kind in any product, service and/or company that could be construed as influencing the position presented in or the review of the manuscript entitled. Written consent statement in their language was completed by all participants.

### ETHICS STATEMENT

The protocols for donkey studies were approved by the Committee on the Ethics of Animal Experiments of Liaocheng University. The ethics approval number is 2023022732.

### PEER REVIEW

The peer review history for this article is available at https://publons.com/publon/10.1002/vms3.1393.

## Data Availability

All relevant data are within the paper.
